# Serum Glycerophospholipid Profile in Acute Exacerbation of Chronic Obstructive Pulmonary Disease

**DOI:** 10.3389/fphys.2021.646010

**Published:** 2021-02-15

**Authors:** Xiaoyan Gai, Chenglin Guo, Linlin Zhang, Lijiao Zhang, Mairipaiti Abulikemu, Juan Wang, Qingtao Zhou, Yahong Chen, Yongchang Sun, Chun Chang

**Affiliations:** Department of Respiratory and Critical Care Medicine, Peking University Third Hospital, Beijing, China

**Keywords:** glycerophospholipids, metabolomics, chronic obstructive pulmonary disease, acute exacerbation of chronic obstructive pulmonary disease, inflammatory subtype

## Abstract

Studies have shown that glycerophospholipids are involved in the pathogenesis of chronic obstructive pulmonary disease (COPD). This study adopted targeted metabolomic analysis to investigate the changes in serum glycerophospholipids in acute exacerbation of chronic obstructive pulmonary disease (AECOPD) and their differential expression in patients with different inflammatory subtypes. Patients with AECOPD admitted between January 2015 and December 2017 were enrolled, and their clinical data were collected. The patients’ gender, age, body mass index, and lung function were recorded. Routine blood and induced sputum tests were performed. Liquid chromatography-mass spectrometry was used to detect the serum glycerophospholipid metabolic profiles and to analyze the metabolic profile changes between the acute exacerbation and recovery stages as well as the differences between different inflammatory subtypes. A total of 58 patients were hospitalized for AECOPD, including 49 male patients with a mean age of 74.8 ± 10.0 years. In the metabolic profiles, the expression of lysophosphatidylcholine (LPC) 18:3, lysophosphatidylethanolamine (LPE) 16:1, and phosphatidylinositol (PI) 32:1 was significantly reduced in the acute exacerbation stage compared to the recovery stage (*P* < 0.05). The three glycerophospholipids were used to plot the receiver operating characteristic curves to predict the acute exacerbation/recovery stage, and the areas under the curves were all above 70%. There were no differential metabolites between the two groups of patients with blood eosinophil percentage (EOS%) ≥2% and <2% at exacerbation. The expression of LPC 18:3, LPE 16:1, and PI 32:1 was significantly reduced in the acute exacerbation stage compared to the recovery stage in the inflammatory subtype with blood EOS <2% (*P* < 0.05). Abnormalities in the metabolism of glycerophospholipids may be involved in the onset of AECOPD, especially in the non-eosinophilic subtype.

## Introduction

Chronic obstructive pulmonary disease (COPD) is characterized by persistent airflow limitation and chronic airway inflammation. It is a common and frequently occurring disease with a global incidence of 10% and has become a worldwide public health problem (Global Initiative for Chronic Obstructive Lung Disease, 2020). The prevalence of COPD in China is 13.7% among people aged 40 years and above, and the total patient population is approximately 100 million (Zurcher et al., 2020). Furthermore, COPD places a substantial disease burden on individuals and families as well as the society and the economy.

Acute exacerbation (AE) of COPD (AECOPD) refers to the exacerbation of any symptoms (e.g., cough, sputum, and wheezing) in COPD patients. It can be caused by bacterial or viral infection, environmental pollution, cold weather, or interruption of routine treatment. It is a leading cause of hospital admission and death (Berenyi et al., 2020). AECOPD often accelerates disease progression, and each AE worsens the patient’s lung function, aggravates complications, and increases the risk of re-hospitalization. Thus, the main goal of COPD treatment is to reduce the frequency of exacerbations. At present, the underlying mechanisms of AECOPD are unclear, and the pathophysiology of the different subtypes is poorly understood. Therefore, metabolomics can be helpful to further investigate the mechanisms and classifications of this disease and explore its biomarkers in greater depth (Kilk et al., 2018; Liang et al., 2019; Ekroos et al., 2020).

Glycerophospholipids are major components of cell membranes, storage materials for bioactive substances, and precursors of informational molecules. They serve important physiological functions in cell growth, migration, signal recognition and transduction, and apoptosis. Glycerophospholipid molecules can be modified by phospholipase A2 to further produce metabolites such as lysophospholipids. Recent studies have shown that glycerophospholipids are involved in the pathogenesis of lung infections (Mander et al., 2002), asthma, and COPD (Telenga et al., 2014; Cruickshank-Quinn et al., 2018; Chen et al., 2019; Gai et al., 2019; Liang et al., 2019) and are associated with the lipid metabolic disorder of alveolar surfactants (Cruickshank-Quinn et al., 2018; Ekroos et al., 2020). However, to date, the differences in the glycerophospholipids profile in the AE stage of COPD and among different inflammatory subtypes have been poorly investigated.

This study aimed to investigate the differences in the serum glycerophospholipid metabolic profiles of AECOPD and among different inflammatory subtypes to provide a basis for further exploring the pathogenesis of AECOPD and identifying precise therapeutic targets for different subtypes.

## Patients and Methods

### Patients

A prospective study was conducted on 58 patients with AECOPD who were admitted to the Department of Respiratory and Critical Care Medicine at Peking University Third Hospital between January 2015 and December 2016.

Inclusion criteria were as follows: (1) age >40 years; (2) COPD diagnosis meeting the GOLD criteria (Global Initiative for Chronic Obstructive Lung Disease, 2020); (3) stable-phase pulmonary function report in the last 6 months; forced expiratory volume in 1 s (FEV_1_)/forced vital capacity (FVC) ratio <70% after bronchodilator inhalation and percentage of predicted FEV_1_ value (FEV1%pred) <80%; and (4) AECOPD meeting the GOLD diagnostic criteria (Global Initiative for Chronic Obstructive Lung Disease, 2020).

Exclusion criteria were as follows: (1) comorbidity with other lung diseases such as active tuberculosis, bronchiectasis, asthma, interstitial lung disease, pleural effusion from various causes, and lung malignancies; and (2) intravenous or oral glucocorticoid therapy for AEs within 28 days.

This study was approved by the Ethics Committee of Peking University Third Hospital (approval number: LM2016032), and written informed consent was provided by the patients enrolled in the study.

### Methods

#### Clinical Data Collection

**FIGURE 1:**
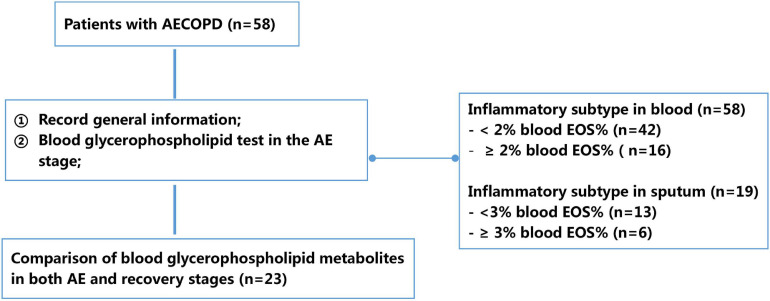
Flowchart of study design. AECOPD, acute exacerbation of chronic obstructive pulmonary disease; EOS, eosinophil percentage.

Demographic information (e.g., gender, age, body mass index, and smoking history), status of inhaled corticosteroids, number of AEs in the past year, and stable-phase lung function parameters in the past 6 months (e.g., FEV1%pred and FEV1/FVC) were recorded for all patients. Patients’ AECOPD symptoms were also recorded, and 2 mL of peripheral blood was collected for serum phospholipid profiling. To further clarify the characteristics of different AECOPD subtypes, serum glycerophospholipid assays were performed on the first day of admission during the AE stage and on the 10–14 days after treatment of AECOPD (before discharge). In addition, patients were classified into different inflammatory subtypes based on whether their peripheral blood eosinophil percentage (EOS%) was ≥2% or <2%, and whether sputum EOS% was ≥3% or <3% to compare the differences in serum glycerophospholipid profiles (Figure 1).

#### Sputum Cytology

Patients were asked to rinse their oral cavity and posterior oropharynx with 3% hypertonic saline prior to sampling. Sputum was then produced spontaneously, or induced sputum was collected. For the collection of induced sputum, patients inhaled an aerosol of 3% saline and coughed up a sufficient amount of sputum within 30 min. The sputum was treated with 0.4% dithiothreitol (DTT, Millipore, Canada), homogenized by shaking at 37°C for 30 min, and stained for cytological examination. The remainder was divided into 0.5 mL portions and stored at −80°C for glycerophospholipid testing (Cao et al., 2006).

#### Serum Glycerophospholipid Profiling

Liquid chromatography-mass spectrometry (LC-MS) was performed to determine serum glycerophospholipid profiles, as described in our previous publications (Gai et al., 2019; Liang et al., 2019). Briefly, the serum was separated from whole blood and lipid extraction was carried out using the Waters Acquity UPLC system, with a UPLC BEH C18 column (1.7 μm; internal diameter, 100 × 2.1 mm). An AB Sciex 5500 QTRAP mass spectrometer was used with the following specifications: ion source, Turbo Ion Spray electrospray ionization; scan mode, multiple reaction monitoring, with the following ion source parameters: CUR = 40 psi, GS1 = 30 psi, GS2 = 30 psi, IS = −4,500 V, CAD = MEDIUM, and TEMP = 350°C. The glycerophospholipid scanning strategy has been described in our previous study (Gai et al., 2019). A total of 14 classes of glycerophospholipids (129 species) were analyzed in this study: phosphatidylcholine (PC, 21 species), alkylphosphatidylcholine [PC(O), 15 species], phosphatidylcholineplasmalogen (PCP, 8 species), phosphatidylethanolamine (PE, 12 species), alkylphosphatidylethanolamine (PEO, 4 species), phosphatidylethanolamine plasmalogen (PEP, 2 species), phosphatidylglycerol (PG, 2 species), phosphatidylinositol (PI, 11 species), lysophosphatidylcholine (LPC, 11 species), lysophosphatidylethanolamine (LPE, 12 species), lysophosphatidylserine (LPS, 5 species), lysophosphatidylglycerol (LPG, 11 species), lysophosphatidylinositol (LPI, 9 species), and lysoalkylphosphatidylcholine (LPCO, 6 species).

### Statistical Methods

The metabolic profiles of glycerophospholipid levels in patients with different COPD subtypes were compared using multivariate statistics (Chong et al., 2018). Partial least squares–discrimination analysis (PLS-DA) was performed for the patients’ samples, and differential metabolites were screened using methods such as estimation of variable importance in projection values, loading weights, and correlation coefficients. The differential metabolites that contributed to subtyping were further subjected to one-way analysis of variance and Bonferroni multiple comparisons to validate the results obtained by above statistics. Data and statistical analyses were performed using the SPSS 19.0 package. Normally distributed measurement data are expressed as $\bar{x}$ ± s, and the χ^2^-test was performed for the comparison of constituent ratios. The intensity of LPC, LPE, and PI were evaluated using the receiver operating characteristic (ROC) curve method. *P* ≤ 0.05 was considered statistically significant.

## Results

### General Information

Fifty-eight patients with AECOPD were included (mean age, 74.8 ± 10.0 years; mean FEV1%pred, 42.6 ± 18.2%; and FEV1/FVC, 49.2 ± 10.4%; Table 1).

**TABLE 1:** General information of patients with AECOPD.

Variable	Value
Male/female	49/9
Age (years)	74.8 ± 10.0
BMI (kg/m^2^)	22.1 ± 4.9
Current smokers	20 (34.5%)
Former smokers	33 (56.9%)
Never smokers	5 (8.6%)
Smoking (pack-years)	39.1 ± 31.1
COPD duration (years)	14.6 ± 12.0
AEs in the past year	1.57 ± 1.36
Concomitant respiratory failure	25 (41.7%)
ICU on admission (%)	11 (19.0%)
Need for non-invasive ventilation on admission (%)	11 (19.0%)
Need for invasive ventilation on admission (%)	2 (3.4%)
FEV_1_%pred	42.6 ± 18.2
FEV_1_/FVC (%)	49.2 ± 10.4
Blood EOS count (/10^9^/L)	0.04 (0.00, 0.14)
Blood EOS ratio (%)	0.50 (0.10, 2.10)

**AECOPD, acute exacerbation of chronic obstructive pulmonary disease; FEV_*l*_, forced expiratory volume in 1 s; FEV_1_%pred, FEV_*l*_ expressed as a percentage of the predicted value; FVC, forced vital capacity; EOS, eosinophil. All data with a normal distribution are shown as the mean ± standard deviation. Non-normally distributed data are expressed as median (25–75%).**

### Metabolic Profile Changes During the AE and Recovery Stages

**FIGURE 2:**
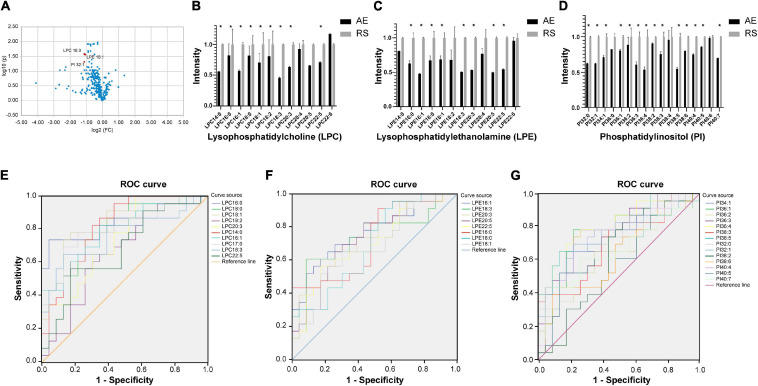
**(A)** Volcano plot showing glycerophospholipids with significant differences between the AE and recovery stages of COPD. The *x*-axis is the logarithm to the base 2 of the ratio of a given phospholipid in the AE and recovery stages, and the *y*-axis is the logarithm to the base 10 of the *P*-value of that phospholipid in different subgroups. The phospholipids that showed significant changes were LPC 18:3, LPE 16:1, and PI 32:1. **(B–D)** Histograms showing all LPC, LPE, and PI in the metabolic profiles between the AE and recovery stages (*P <0.05). AE, acute exacerbation; RS, recovery stage. **(E–G)** ROC curve analysis of the main differential glycerophospholipids during COPD recovery (LPC, LPE, and PI). ROC curve analysis was performed using the main differential glycerophospholipids above as the test variables and the recovery phase as the state variable. The areas under the curve (AUCs) for LPC 14:0, 16:0, LPC 16:1, LPC 17:0, LPC 18:0, LPC 18:1, LPC 18:2, LPC 18:3, LPC 20:3, and LPC 22:5 were 82.0, 84.5, 79.6, 80.5, 80.7, 83.0, 66.7, 76.4, 74.5, and 68.1%, respectively. The AUCs of LPE 16:0, LPE 16:1, LPE 18:0, LPE 18:1, LPE 18:3, LPE 20:3, LPE 20:5, and LPE 22:5 were 72.4, 76.4, 69.2, 67.9, 73.5, 73.2, 72.4, and 70.1%, respectively. The AUCs of PI 32:0. PI 32:1, PI 34:1, PI 36:1, PI 36:2, PI 36:3, PI 36:4, PI 38:2, PI 38:3, PI 38:5, PI 38:6, PI 40:4, PI 40:5, and PI 40:7 were 72.6, 74.3, 75.4, 75.0, 63.9, 78.4, 81.1, 61.4, 69.8, 80.2, 62.6, 74.1, 63.1, and 66.0%, respectively.

Among the 58 patients included, serum was collected from 23 patients to compare the glycerophospholipid metabolic profiles between the AE and recovery (7–10 days after admission) stages. PLS-DA indicated that the global glycerophospholipids tends to be distinguishable from the metabolic profiles of AE and recovery stages. The volcano plots (Figure 2A) showed that there were three differential glycerophospholipid metabolites after false discovery rate (FDR) correction with significant changes: LPC 18:3, LPE 16:1, and PI 32:1. Table 2 shows the names and fold changes of the differential metabolites identified after data comparison (*P* <0.05). Figures 2B–D show the histograms of all LPC, LPE, and PI species in the metabolic profiles obtained between the AE and recovery stages (^∗^*P* <0.05). An ROC curve analysis was performed with the three most significantly differential glycerophospholipids as the test variables and recovery stages as the state variable. The area under the curves (AUCs) for LPC 14:0, LPC 16:0, LPC 16:1, LPC 17:0, LPC 18:0, LPC 18:1, LPC 18:2, LPC 18:3, LPC 20:3, and LPC 22:5 were 82.0, 84.5, 79.6, 80.5, 80.7, 83.0, 66.7, 76.4, 74.5, and 68.1%, respectively. The AUCs of LPE 16:0, LPE 16:1, LPE 18:0, LPE 18:1, LPE 18:3, LPE 20:3, LPE 20:5, and LPE 22:5 were 72.4, 76.4, 69.2, 67.9, 73.5, 73.2, 72.4, and 70.1%, respectively. The AUCs of PI 32:0. PI 32:1, PI 34:1, PI 36:1, PI 36:2, PI 36:3, PI 36:4, PI 38:2, PI 38:3, PI 38:5, PI 38:6, PI 40:4, PI 40:5, and PI 40:7 were 72.6, 74.3, 75.4, 75.0, 63.9, 78.4, 81.1, 61.4, 69.8, 80.2, 62.6, 74.1, 63.1, and 66.0%, respectively.

**TABLE 2:** List of differential metabolites in the AE and recovery stages.

Metabolites	FC (AE/recovery)	log2 (FC)	Corrected *P*-value
LPC 16:0	1.226	0.294	0.011
PI 36:4	1.860	0.895	0.012
LPC 14:0	1.786	0.837	0.012
LPC 16:1	1.765	0.819	0.012
LPC 17:0	1.425	0.511	0.012
LPC 18:1	1.423	0.509	0.012
LPC 18:0	1.229	0.298	0.012
PI 36:3	1.644	0.717	0.014
PI 38:5	1.825	0.868	0.021
LPC 18:3	2.166*	1.115	0.027
LPE 16:1	2.093*	1.066	0.027
PI 34:1	1.402	0.487	0.027
LPC 20:3	1.585	0.664	0.041
PI 32:1	2.178*	1.123	0.048

*Three main differential metabolites analyzed using a fold change of two.

*AE, acute exacerbation; FC, fold change; LPC, lysophosphatidylcholine; LPE, lysophosphatidylethanolamine; PI, phosphatidylinositol.*

### Differences in the Metabolic Profiles of Patients With Different Inflammatory Subtypes During the AE Stage

Routine blood tests were conducted for 58 patients with AECOPD; the results showed that blood eosinophils accounted for <2 and ≥2% of the total white blood cells in 42 and 16 patients, respectively (Supplementary Table 1). PLS-DA and FDR correction indicated that there were no differential metabolites between the two groups of patients with peripheral blood EOS% ≥2 and <2%.

Nineteen patients also underwent sputum examination: based on sputum cell counts, 6 patients were classified as sputum eosinophilic (≥3%) and 13 as non-eosinophilic (<3%) types. PLS-DA showed differences in phospholipid metabolic profiles between the two groups, but no significantly differential metabolites were identified after FDR correction.

### Changes in Metabolic Profiles Between AE and Recovery Stage of Different Inflammatory Subtypes

**FIGURE 3:**
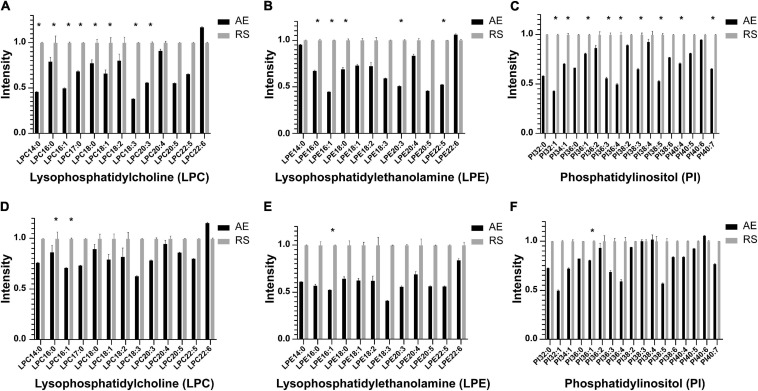
Histogram showing LPC, LPE, and PI species in the metabolic profiles between the AE and recovery stages in different inflammatory subtypes. **(A–C)** In patients with EOS% <2%, the concentrations of LPC 14:0, LPC 16:0, LPC 16:1, LPC 18:0, LPC 18:1, LPC 18:3, LPC 20:3, LPE 16:0, LPE 16:1, LPE 18:0, LPE 20:3, LPE 22:5, PI 32:1, PI 34:1, PI 36:1, PI 36:3, PI 36:4, PI 38:3, PI 38:5, PI 40:4, and PI 40:7 significantly decreased in the AE stage compared to the recovery stage (*P < 0.05). **(D–F)** In patients with EOS% > 2%, only the concentrations of LPC 14:0, LPC 16:1, LPE 16:1, and PI 36:1 were significantly decreased in the AE stage compared to the recovery stage (*P < 0.05).

Among the 58 patients included, serum was collected from 23 patients to compare the glycerophospholipid metabolic profiles between the AE and recovery stages, including 8 patients with blood EOS ≥ 2% and 15 patients with EOS% < 2%. In patients with EOS% < 2%, the concentrations of LPC 14:0, LPC 16:0, LPC 16:1, LPC 18:0, LPC18:1, LPC18:3, LPC 20:3, LPE 16:0, LPE 16:1, LPE 18:0, LPE 20:3, LPE 22:5, PI 32:1, PI 34:1, PI 36:1, PI 36:3, PI 36:4, PI 38:3, PI 38:5, PI 40:4, and PI 40:7 were significantly lower in the AE stage than in the recovery stage (^∗^*P* < 0.05) (Figures 3A–C). In patients with EOS% > 2%, only the concentrations of LPC 14:0,LPC 16:1, LPE 16:1, and PI 36:1 were significantly lower in the AE stage than in the recovery stage (^∗^*P* < 0.05) (Figures 3D–F). These findings indicated that the changes in LPC, LPE, and PI between the AE and recovery stages were mainly manifested in AECOPD patients with EOS% < 2%.

## Discussion

COPD is a highly heterogeneous disease presenting with different phenotypes. In this study, metabolomic analysis of glycerophospholipids was conducted among patients in the acute and recovery stages of COPD and also for different inflammatory subtypes, involving 129 phospholipids from 14 classes. Comparison of the metabolic profiles between the AE and recovery stages of COPD revealed significant decreases in LPC18:3, LPE 16:1, and PI 32:1 levels, suggesting the involvement of abnormal glycerophospholipid metabolism in the onset of AECOPD.

Glycerophospholipids are important components of the cell membrane and were thought to be related to cell structure and storage compartments. However, it has recently been discovered that glycerophospholipids can participate in mediating airway inflammation through its cleavage by phospholipase A2 to produce lysophospholipids and, hence, become involved in signaling and immune responses. Lysophospholipids are monoacyl hydrolysates of diacyl glycerophospholipid precursor molecules and are so called due to their detergent-like ability to lyse erythrocytes (Makide et al., 2014). In addition to altering the physical structure of cellular lipid bilayers, lysophospholipids can regulate cell signaling pathways by binding directly to membrane G-protein-coupled receptors and indirectly affecting membrane receptors. The main physiological lysophospholipids include LPA, LPS, LPG, and LPC. Recent studies have found that glycerophospholipids are physiologically active intercellular and intracellular lipid mediators involved in the pathogenesis of lung infections (Mander et al., 2002), asthma, and COPD (Sevastou et al., 2013; Drzazga et al., 2014; Telenga et al., 2014; Cruickshank-Quinn et al., 2018; Chen et al., 2019).

LPC is a lysophospholipid with a relatively high concentration in human blood and is produced by the hydrolysis of PC by phospholipase A2; conversely, LPE is produced by the hydrolysis of phosphatidylethanolamine by phospholipase A2. Based on our study results, lipid metabolites, such as LPC, LPE, and PI, were significantly reduced in AECOPD patients. Cigarette smoking is a major cause of COPD. Glycerophospholipids, which are complexes composed of 90% phospholipids and a small amount of protein, are important components of lung surfactants, which may be damaged following smoke exposure (Schurch et al., 1992; Goerke, 1998; Berry et al., 2011). They are secreted into the alveoli by airway epithelial cells to reduce alveolar surface tension and block pathogen invasion. Cruickshank-Quinn et al. used LC-MS to study smokers with COPD and found abnormal expression of several serum glycerophospholipids, among which LPC and PI were significantly negatively correlated with FEV_1_%pred and FEV_1_/FVC; moreover, they also performed integrated transcriptome/metabolome analysis and showed that the expression of LPC, PI, PE, and LPE gradually decreased with worsening lung function outcomes (Cruickshank-Quinn et al., 2018). Halper-Stromberg et al. (2019) performed LC-MS analysis of bronchoalveolar lavage fluid (BALF) from COPD patients and showed that the concentrations of lipid metabolites, such as LPC, LPE, and PI, were significantly reduced in COPD patients, and that these metabolites negatively correlated with FEV1/FVC. Furthermore, the level of lipid metabolites in BALF correlated with COPD outcomes more closely than that of serum metabolites. These findings suggest that LPC, LPE, and PI are associated with COPD, which is consistent with our results. The lipid metabolism disorder involving alveolar surfactants in patients with COPD (Cruickshank-Quinn et al., 2018; Ekroos et al., 2020) may be a potential therapeutic target for future studies on the restoration of alveolar surfactants. Exposure to cigarette smoke can induce alveolar surfactant dysfunction, alveolar epithelial cell apoptosis, and emphysema. These manifestations may be particularly prominent during AEs. Fatty acids undergo dynamic transformations in the body. The acyl group at the sn-2 site of PC is subjected to continuous deacylation and reacylation, leading to its repeated inversion and re-modification in a process called the Lands cycle (Wang and Tontonoz, 2019). PC is hydrolyzed by phospholipase to form LPC, and lysophosphatidylcholine acyltransferase (LPCAT) is a key enzyme of the Lands cycle. LPCAT is involved in catalyzing the conversion of LPC and acyl-CoA into PC and free CoA, thereby reducing the concentration of endogenous LPC (Wang and Tontonoz, 2019). COPD patients have a high expression of *LPCAT* gene, which correlates with the severity of FEV_1_%pred lung function (Cruickshank-Quinn et al., 2018). In addition, chronic airway inflammatory diseases, such as COPD, often involve abnormalities in phospholipase metabolism, which contribute to the differences in phospholipid expression (Wymann and Schneiter, 2008; Azimzadeh Jamalkandi et al., 2015).

*In vitro* studies have shown that LPC may exhibit pro-inflammatory or anti-inflammatory physiological effects in different pathophysiological conditions. As a representative of pro-inflammatory lysophospholipids, LPC is involved in regulating T-cell function and immune activity, inducing the processing and secretion of IL-1β, and increasing the expression of cytokine-induced interferon gamma (IFN-γ) and transforming growth factor β1 (TGF-β1) (Drzazga et al., 2014). In addition, LPC-dependent NADPH oxidase can stimulate the production of reactive oxygen species, which promotes the conversion of pro-cytokines to their mature, biologically active forms (IL-1β, IL-18, and IL33) (Schilling and Eder, 2010). Furthermore, in addition to their pro-inflammatory effects, polyunsaturated LPCs, such as LPC 20:4, LPC 20:5, and LPC 22:6, can act as potent anti-inflammatory agents against the activity of immune responses induced by saturated LPC (Riederer et al., 2010; Hung et al., 2012). LPCs can also downregulate the formation of pro-inflammatory mediators (IL-5, IL-6, NO, 12-hydroxyeicosatetraenoic acid, and LPC16:0-induced PGE2) and upregulate the expression of anti-inflammatory mediators (IL-4 and IL-10) by reducing leukocyte extravasation and plasma leakage, thereby achieving anti-inflammatory effects (Hung et al., 2012). Thus, the different biological activities of LPCs are related to the body’s internal environment, including hypoxia, oxidative stress, T-cell immune homeostasis, and differences in phospholipase metabolism (Murakami et al., 2014); importantly, these activities can vary with the length and saturation of the acyl chain, which also affects the biological properties and activity of the resultant molecules (Drzazga et al., 2014). Our results showed that LPC was decreased in AECOPD, and the mechanism underlying this reduction deserves further study.

Our study showed that LPE was also lower in the AE stage compared to the recovery stage, but the exact mechanism is unclear. Hung et al. found that unsaturated LPE has anti-inflammatory effects (Hung et al., 2011). In their study, zymosan A was used to induce acute peritonitis in mice, and treatment with polyunsaturated acyl-LPE could effectively reduce peritoneal inflammation in mice, while also reducing the formation of LTC4, which is a lipid mediator involved in vascular permeability. Furthermore, the levels of pro-inflammatory mediators (IL-1b, IL-6, TNF-α, and NO) decreased, whereas those of the anti-inflammatory mediator IL-10 increased. Taken together, these results suggest that LPE may have an anti-inflammatory effect. In addition, lysophosphatidyl transferase may be a regulatory factor (Eto et al., 2020). AECOPD is a complex and systemic disease state, and the mechanisms underlying the action of LPC, LPE, and PI in COPD deserve further investigation.

Different inflammatory subtypes can be identified in AECOPD (Yun et al., 2018). The present study defined the eosinophilic subtype as the ratio of peripheral blood EOS to leukocytes ≥ 2% and sputum EOS ≥ 3%. We found that COPD patients with the peripheral blood eosinophilic subtype had a shorter duration of COPD, lower rate of concomitant respiratory failure on admission, lower rate of non-invasive ventilation during hospitalization, shorter hospital stay, and fewer AEs in the past year than the sputum EOS < 3% subtype. This finding suggests that there may be a correlation between peripheral blood EOS levels and AECOPD severity in COPD patients (Kostikas et al., 2018). However, this study did not find significant differences in the levels of glycerophospholipid metabolites between the inflammatory subtypes, which may be due to the small sample size. Nevertheless, the results showed that the changes in LPC, LPE, and PI between AE and the recovery stage were more significant in subtypes with blood EOS% < 2% than in those with blood EOS% > 2%. This result indicates that LPC, LPE, and PI may play a role in non-eosinophilic COPD and warrants further study.

This study has some limitations. First, this study had a small sample size since it was primarily an exploratory study. Second, the peripheral blood glycerophospholipid levels measured in this study is a systemic response to chronic airway inflammation and cannot fully represent the local environment in the bronchi and lungs. The next step is to increase the sample size, combine sputum and BALF specimen analysis to further verify its relationship with AECOPD, and conduct in-depth mechanistic studies.

In conclusion, the present metabolomics study used LC-MS to detect the metabolic profiles of serum phospholipids in AECOPD and different subtypes of COPD. LPC, LPE, and PI were significantly reduced in AECOPD and could be used as biomarkers and potential therapeutic targets for treating AECOPD. Metabolomic analysis of glycerophospholipids may become an important research tool that could give rise to new drug targets and new biomarkers for COPD subtypes. Thus, the treatment of AECOPD is expected to evolve rapidly toward phenotypic specificity and individualization.

## Data Availability Statement

The original contributions presented in the study are included in the article/Supplementary Material, further inquiries can be directed to the corresponding author/s.

## Ethics Statement

The studies involving human participants were reviewed and approved by the Ethics Committee of Peking University Third Hospital. The patients/participants provided their written informed consent to participate in this study.

## Author Contributions

XG, CG, and CC participated in the acquisition, analysis, and interpretation of the data. XG wrote the report. All authors contributed to this article and reviewed and approved the final version.

## Conflict of Interest

The authors declare that the research was conducted in the absence of any commercial or financial relationships that could be construed as a potential conflict of interest.
