# Supplementary material for: Serum Glycerophospholipid Profile in Acute Exacerbation of Chronic Obstructive Pulmonary Disease
Source: Front Physiol. 2021 Feb 15;12:646010. doi: 10.3389/fphys.2021.646010 (PMC7917046; doi:10.3389/fphys.2021.646010)
Supplement: Supplementary file 1 [file Table_1.DOCX]

| **Supplementary Table**  **Table 1. Comparison of Clinical Characteristics between Patients with Different Inflammatory Subtypes** | | | | |  |
| --- | --- | --- | --- | --- | --- |
|  | EOS<2.0%(n= 42) | EOS≥2.0%(n= 16) | *P* value |  |  |
| Male/Female | 35/7 | 13/3 | 1.000 |  |  |
| Age (years) | 74.3 ± 10.5 | 76.0 ± 8.7 | 0.569 |  |  |
| BMI（kg/m^2^） | 22.2 ± 5.3 | 21.9 ± 3.8 | 0.838 |  |  |
| Current smokers | 14 (33.3%) | 6 (37.5%) | 0.893 |  |  |
| Former smokers | 25 (59.5%) | 8 (50.0%) |  |  |  |
| Never smoked | 5 (11.9%) | 2 (12.5%) |  |  |  |
| Smoking (pack-years) | 35.7±28.2 | 48.4±37.2 | 0.163 |  |  |
| COPD duration (years) | 16.7±11.9 | 8.9±10.5 | 0.023 |  |  |
| AEs in the past year | 1.77±1.48 | 1.00±0.73 | 0.010 |  |  |
| Time from AE to consultation (days) | 12.8±14.3 | 23.5±28.5 | 0.170 |  |  |
| Repeated AE during follow-up | 12 (28.5%) | 3 (18.8%) | 0.738 |  |  |
| Time to next AE (month) ^*^ | 4.7±3.8 | 9.0±4.4 | 0.113 |  |  |
| Concomitant respiratory failure | 24 (57.1%) | 1 (6.3%) | 0.001 |  |  |
| ICU on admission (%) | 11 (25.0%) | 1 (6.3%) | 0.153 |  |  |
| Non-invasive ventilation on admission (%) | 10 (26.2%) | 1 (6.3%) | 0.010 |  |  |
| Invasive ventilation on admission (%) | 2 (4.8%) | 0 (0.0%) | 1.000 |  |  |
| FEV_1_% | 40.4±19.5 | 49.1±12.0 | 0.192 |  |  |
| FEV_1_/FVC (%) | 48.0±10.9 | 52.7±7.8 | 0.213 |  |  |
| Blood EOS count (/10^9^/L) | 0.09±0.29 | 0.25±0.19 | 0.036 |  |  |
| Systemic corticosteroid therapy | 24 (57.1%) | 3 (18.8%) | 0.014 |  |  |
| Previous long-term ICS treatment (%) | 20 (47.6%) | 4 (25.0%) | 0.342 |  |  |
| Non-invasive ventilation during  hospitalization (%) | 9 (21.4%) | 0 (0.0%) | 0.050 |  |  |
| Hospitalization duration (days) | 15.2±7.4 | 10.9±5.0 | 0.034 |  |  |
| ^*^:Acute exacerbation occurred again in 15 patients during follow-up  Note: AE, Acute exacerbation; FEV_l_, forced expiratory volume in 1 second; FEV_1_%pred, FEV_l_ expressed as a percentage of the predicted value; FVC, forced vital capacity. All data with a normal distribution are shown as the mean ± standard deviation. Non-normally distributed data are expressed as median (25%–75%). | | | | |  |
